# First Detection of Tetrodotoxin in Greek Shellfish by UPLC-MS/MS Potentially Linked to the Presence of the Dinoflagellate *Prorocentrum minimum*

**DOI:** 10.3390/toxins7051779

**Published:** 2015-05-20

**Authors:** Aristidis Vlamis, Panagiota Katikou, Ines Rodriguez, Verónica Rey, Amparo Alfonso, Angelos Papazachariou, Thetis Zacharaki, Ana M. Botana, Luis M. Botana

**Affiliations:** 1 Department of Pharmacology, Veterinary School, University of Santiago de Compostela, Lugo 27002, Spain; E-Mails: aristidis.vlamis@rai.usc.es (A.V.); ines.rodriguez@usc.es (I.R.); veronica.rey@rai.usc.es (V.R.); amparo.alfonso@usc.es (A.A.); anamaria.botana@usc.es (A.M.B.); luis.botana@usc.es (L.M.B.); 2 National Reference Laboratory on Marine Biotoxins, Veterinary Centre of Thessaloniki, Ministry of Productive Reconstruction, Environment and Energy, 3A Limnou street, GR 54627 Thessaloniki, Greece; E-Mails: aopvet@gmail.com (A.P.); thetisz@otenet.gr (T.Z.)

**Keywords:** Aegean Sea, emerging biotoxins, Mediterranean Sea, cultured mussels, *Mytilus galloprovincialis*, *Prorocentrum minimum*, tetrodotoxin, toxic episode, UPLC-MS/MS, venerupin shellfish toxin

## Abstract

During official shellfish control for the presence of marine biotoxins in Greece in year 2012, a series of unexplained positive mouse bioassays (MBA) for lipophilic toxins with nervous symptomatology prior to mice death was observed in mussels from Vistonikos Bay–Lagos, Rodopi. This atypical toxicity coincided with (a) absence or low levels of regulated and some non-regulated toxins in mussels and (b) the simultaneous presence of the potentially toxic microalgal species *Prorocentrum minimum* at levels up to 1.89 × 10^3^ cells/L in the area’s seawater. Further analyses by different MBA protocols indicated that the unknown toxin was hydrophilic, whereas UPLC-MS/MS analyses revealed the presence of tetrodotoxins (TTXs) at levels up to 222.9 μg/kg. Reviewing of official control data from previous years (2006–2012) identified a number of sample cases with atypical positive to asymptomatic negative MBAs for lipophilic toxins in different Greek production areas, coinciding with periods of *P. minimum* blooms. UPLC-MS/MS analysis of retained sub-samples from these cases revealed that TTXs were already present in Greek shellfish since 2006, in concentrations ranging between 61.0 and 194.7 μg/kg. To our knowledge, this is the earliest reported detection of TTXs in European bivalve shellfish, while it is also the first work to indicate a possible link between presence of the toxic dinoflagellate *P. minimum* in seawater and that of TTXs in bivalves. Confirmed presence of TTX, a very heat-stable toxin, in filter-feeding mollusks of the Mediterranean Sea, even at lower levels to those inducing symptomatology to humans, indicates that this emerging risk should be seriously taken into account by the EU to protect the health of shellfish consumers.

## 1. Introduction

Tetrodotoxin (TTX) is an extremely potent neurotoxin that can block sodium channels and thus inhibit propagation of action potentials in muscle and nerve cells [1]. Named after the *Tetraodontidae* puffer fish family, TTX is perhaps most notorious as the toxin that causes puffer fish poisoning. TTX can cause death by muscular paralysis, respiratory depression and circulatory failure [2]. The minimum lethal and minimum acute doses of TTX to human (wt. 50 kg) are estimated to be around 2 mg and 0.2 mg, respectively. Symptoms usually appear within 10–45 min of exposure, depending upon the amount of the toxin ingested; however, some of the reported cases were asymptomatic until as much as 3 to 6 h after exposure. The initial symptom is usually oral paresthesia, which gradually spreads to the extremities and trunk. Other early symptoms comprise taste disturbance, dizziness, headache, diaphoresis, and pupillary constriction, potentially accompanied by gastrointestinal symptoms of salivation, hypersalivation, nausea, vomiting, hyperemesis, hematemesis, hypermotility, diarrhea and abdominal pain [3,4].

Tetrodotoxin is known to occur in a variety of fish species and other organisms including arthropods, echinoderms, an alga, mollusks, worms, newts, frogs and toads [5,6,7,8,9]. To date, however, reported occurrences of TTX in bivalve mollusks (clams, cockles, mussels, oysters, scallops and others) are very few. TTX has been determined in New Zealand *Paphies australis* clams [10] and in Japanese scallops [11]. Similarly, reported occurrences in European seafood are very limited. TTX was first detected in 2007, in the course of a non-fatal human intoxication following consumption of contaminated sea snails *Charonia lampas lampas* (a gastropod) harvested in Spain [12]. Very recently, TTX presence has been reported in bivalve mollusks (mussels and Pacific oysters) harvested from the south coast of England, along the Channel [13].

Due to the wide TTX distribution among genetically-unrelated animals, combined with individual, regional and seasonal variation in TTX levels, the exact origin of TTX in the food chain has been long debated, but still the source of the toxin remains practically unknown [5,14]. In contrast to the rest of biotoxins that accumulate in fishery products, TTX production has not been linked until now to any microalgal organism. Contribution of symbiotic bacteria has been suggested to play a role in TTX genesis for marine animals [15]. Such symbiotic bacteria, including *Shewanella* algae, *S. putrefaciens*, *Vibrio* sp., *Pseudomonas* sp., and *Alteromonas tetraodonis*, are suggested to accumulate in the subcutaneous mucus, or in the intestine, releasing the TTX [16,17]. This was later confirmed by the isolation of TTX-producing bacteria from different TTX-bearing animals [13,18,19].

*Prorocentrum minimum* (Pavillard) Schiller (previously reported also as *Exuviaella mariae-lebouriae* Parke and Ballantine and *P. mariae-lebouriae*), a phytoplanctonic dinoflagellate known to cause red tides, is widely distributed in the seas and oceans of the northern hemisphere. *P. minimum* is generally considered harmless; however, episodes of shellfish and human poisoning have been so far reported in Japan, Portugal and Norway. In Japan, a mass-poisoning incident occurred at Lake Hamana in March 1942 after consumption of short-necked clams (*Venerupis semidecussata*), causing death in 114 out of 324 persons affected [20]. A total of seventy-one deaths in the same region were later attributed to ingestion of toxic oysters (*Crassostrea gigas*) in March 1943 and of toxic clams in March 1949. Symptoms included heavy liver injury (necrosis and fatty degeneration), hemorrhagic diathesis with frenzy, unconsciousness and coma, and death occurring within 24–48 h of symptoms initiation [21]. *P. minimum* (*E. mariae-lebouriae*) was attributed as the toxin source, after establishing a relationship between shellfish toxicity and seawater abundance of these dinoflagellates [20,22,23,24]. A nitrogenous toxic substance, which produced symptoms in mice similar to those observed in humans, was isolated by Akiba and Hattori [25] from the mid-gut of the short-necked clam. This substance was named “venerupin” and the associated syndrome was assigned as Venerupin Shellfish Poisoning (VSP) [21]. Much later, *P. minimum* (identified initially as *Exuviaella baltica* and later as *Prorocentrum balticum*) was incriminated for a number of human poisoning episodes in Portugal (Obidos Lagoon) following shellfish consumption. The symptoms were characterized as resembling those of paralytic shellfish poisoning (PSP) [26,27,28]. Reported events with PSP-like symptomatology in the Zhengjiang and Fujiang provinces from 1978 to 1985 were also attributed to *P. minimum*, but details are scarce [29]. Similarly, *P. minimum* was identified as the cause in a case of poisoning after mussels consumption in the Oslofjord, Norway in 1979, as it had bloomed several weeks before in the mussel harvesting area. The symptoms (especially nausea and late gastrointestinal disorders) resembled in nature and development to those of VSP. Preliminary toxin bioassays on mussels sampled from the fjord showed signs of toxicity, with a symptomatology very similar to that of VSP, but less severe [30,31]. Toxin presence has been occasionally investigated at the same time with a *P. minimum* bloom. Kimor *et al.* [32], for instance, failed to find any toxicity in Baltic Sea dinoflagellates, whereas mouse tests revealed only very minor symptoms of toxicity in Black Sea mussels [33]. Red tides caused by *P. minimum* or *E. baltica* (actually *P. minimum*) have also been associated with various effects on marine organisms. Blooms were regarded as highly toxic, since fish and other marine animals died or were forced to flee [27,34,35]. In one case, however, these deaths occurred three days after reduction of dissolved oxygen [34]. It should be noted that in both of these cases, further studies were not undertaken to clarify the source of toxicity.

Effects of algal blooms on shellfish and aquaculture have been reviewed by Shumway [36], who suggested that toxic effects have been often associated with *P. minimum* blooms and that the toxin accumulated in shellfish was able to affect both shellfish harvested from bloom water and humans consuming the shellfish. So far, however, the only unambiguous link between blooms of *P. minimum* and shellfish toxicity has been documented by Grzebyk *et al.* [37] in a French Mediterranean site. In that study, a number of *P. minimum* axenic clones, producing a water-soluble neurotoxic component that rapidly killed mice, were isolated. Prior to that, mussel toxicity had been detected in the Sete region on the French Mediterranean coast during a large bloom of *P. minimum* and *P. micans* [37]. The symptomatology observed in mouse bioassays indicated a rapid neurological effect; however, it was not possible to provide clear evidence that the dinoflagellates were actually the source of this toxicity. Subsequently though, Denardou-Queneherve *et al.* [38] documented the same toxic fraction obtained from shellfish during a large *P. minimum* bloom (56 × 10^6^ cells/L), which occurred in the Salses-Leucate lagoon on the French Mediterranean coast. Despite the fact that this research group provided a substantial contribution towards clarification of the *P. minimum* toxin properties, the nature and structure of the toxin was never identified.

During official shellfish control for the presence of marine biotoxins in Greece in year 2012, a series of unexplained positive mouse bioassays (MBA) for lipophilic toxins with nervous symptomatology prior to mice death was observed in mussels from Vistonikos Bay–Lagos, Rodopi. This atypical toxicity coincided with the absence or presence in trace levels of all known lipophilic, PSP and ASP group toxins in mussels, as well as with the presence of *P. minimum* at levels up to 1.89 × 10^3^ cells/L in the area’s seawater, whereas further analyses by different MBA protocols indicated that the unknown toxin was of hydrophilic nature. In this context, the purpose of this work was: (a) to investigate this episode with regard to the nature of the toxin(s) causing this atypical toxicity and (b) to investigate toxin profiles of shellfish from previous years (2006–2012) from different Greek production areas, collected in time periods when *P. minimum* was present at high abundances.

## 2. Results

### 2.1. The 2012 P. minimum Episode in Vistonikos Bay–Lagos, Rodopi, Greece

#### 2.1.1. Episode History and Preliminary Investigation

**Figure 1:**
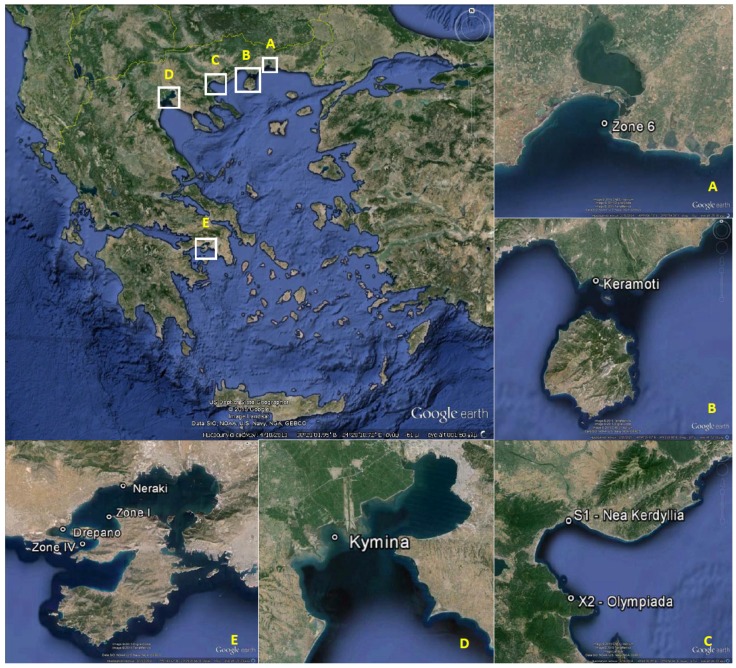
Maps showing locations of sampling stations: (**A**) Vistonikos Bay–Lagos (Rodopi); (**B**) Keramoti Bay (Kavala); (**C**) Strymonikos Bay (Serres, Chalkidiki); (**D**) Thermaikos Gulf (Thessaloniki); and (**E**) Saronikos Gulf (Dytiki Attiki/Megara, Attiki/Nisson). Round marks (•) depict the location of sampling stations.

In late spring-early summer of 2012, a series of four consecutive mussel samples (1770/2012; 1774/2012; 1786/2012 and 1798/2012) derived from the sampling site Zone 6, Vistonikos Bay–Lagos (regional unit of Rodopi, Greece; Table 1, Figure 1A) tested positive in the Yasumoto 1978 (Yas’78) protocol for lipophilic toxins [39], presenting an atypical nervous symptomatology prior to mice death, whereas all four tested negative to the MBA for PSP toxins and below the limit of detection (0.1 mg/kg) for the presence of ASP toxins. During the same time period, phytoplankton counts for all known species associated with the production of regulated marine biotoxin groups (lipophilic, paralytic and amnesic toxins) were all either undetectable or present at low levels not adequate to account for any shellfish toxicity. The only microalgal species present at considerable concentrations was *Prorocentrum minimum* (syn. = *P. cordatum*, as the currently taxonomically accepted name; [40]) reaching levels up to 1.89 × 10^3^ cells/L (see Table 2).

**Table 1:** Locations of sampling stations included in the study for each coastal area expressed by geographical coordinates (longitude and latitude).

Coastal Area	Regional Unit	Sampling Station	Latitude (N)	Longitude (E)
Vistonikos Bay–Lagos	Rodopi	Zone 6	40°58'23.73"	25°07'06.56"
Keramoti Bay	Kavala	Keramoti	40°52'00.70"	24°40'34.60"
Strymonikos Bay	Serres	S1—Nea Kerdyllia	40°46'46.49"	23°49'50.00
	Chalkidiki	X2—Olympiada	40°33'00.60"	23°50'00.00"
Thermaikos Gulf	Thessaloniki	Kymina	40°30'00.22"	22°41'00.06"
Saronikos Gulf	Attiki/Nisson	Zone I—Vasilika–Faneromeni	37°59'42.29"	23°27'50.06"
		Zone IV—Agios Georgios	37°58'09.89"	23°25'53.92"
	Dytiki Attiki/Megara	Neraki	38°01'30.19"	23°28'52.79"
		Drepano	37°59'01.02"	23°24'30.09"

**Table 2:** Sampling data and analysis results of the 2012 *P. minimum* episode in Vistonikos Bay–Lagos, Rodopi, Greece.

Sampling data					*Prorocentrum minimum* (cells/L)	MBA (Yasumoto1978 [39])		MBA (Other)			UPLC-MS/MS				
Sample code	Date of collection	Regional Unit	Site	Species		Result	Symptomatology	Protocol	Result	Symptomatology	Tissue tested	TTX (μg/kg)	4-epi-TTX (μg/kg)	4,9-anhydro-TTX (μg/kg)	Sum of TTXs (μg/kg)
1727/2012	7 May 2012	Rodopi	Zone 6	Mussels	Presence	Negative (− − −)	No symptomatology	N/A	N/A	N/A	DG	46.2	ND	ND	46.2
1770/2012	22 May 2012	Rodopi	Zone 6	Mussels	8.26× 10^2^	Positive (+ + +)	3/3 mice dead in 1:00–1:30 h	CRL-MBA-SOP v.5 (DEE fraction)	Negative (− − −)	None	DG	202.9	7.6	<LOQ (12.4) *	222.9
											WF	179.1	<LOQ (4.2) *	<LOQ (10.3) *	193.6
1774/2012	28 May 2012	Rodopi	Zone 6	Mussels	1.78 × 10^3^	Positive (+ + +)	3/3 mice dead in 1:30–2:00 h	CRL-MBA-SOP v.5 (DEE fraction)	Negative (− − −)	2/3 mice with mild diarrhea	DG	185.9	<LOQ (4.5) *	<LOQ (15.9) *	206.3
1786/2012	30 May 2012	Rodopi	Zone 6	Mussels	1.70 × 10^3^	Positive (+ + +)	3/3 mice dead (1/3 in 1:15–1:30 h, 1/3 in 1:48 h & 1/3 in 1:51 h)	CRL-MBA-SOP v.5 (DEE fraction)	Negative (− − −)	1/3 mice with mild diarrhea	DG	180.0	<LOQ (6.8) *	<LOQ (14.5) *	201.4
								CRL-MBA-SOP v.5 (aqueous fraction)	Positive (+ + +)	3/3 mice dead in 7–10 min					
1798/2012	6 Jun 2012	Rodopi	Zone 6	Mussels	1.89 × 10^3^	Positive (+ + +)	3/3 mice dead (1/3 in 1:31 h, 1/3 in 2:30 h & 1/3 in 2:31)	CRL-MBA-SOP v.5 (DEE fraction)	Negative (− − −)	None	DG	186.2	15.6	<LOQ (14.7) *	216.6
1801/2012	11 Jun 2012	Rodopi	Zone 6	Mussels	5.65 × 10^2^	Negative (− − −)	2/3 mice with diarrhoea	CRL-MBA-SOP v.5 (ether fraction)	Negative (− − −)	1/3 mice with diarrhea	DG	56.5	8.8	22.9	88.2

MBA = Mouse bioassay, TTX = tetrodotoxin, DG = digestive glands, WF = whole flesh, DEE = diethylether, N/A = not applicable, ND = not detected. Values with an asterisk (*) indicate concentrations between the calculated method LOD and LOQ and are shown as contributions to the sum of TTXs.

According to the documented procedures of the Greek National Reference Laboratory on Marine Biotoxins (NRLMB) at that time and due to the fact that Regulation (EC) 15/2011 [41] was already in place, according to which regulatory monitoring decisions with regard to lipophilic toxins have to be based on the results of the LC-MS/MS method (EU-Harmonised SOP-LIPO-LC-MS/MS, Version 4, European Reference Laboratory for Marine Biotoxins, Vigo, Spain) in case of conflict, samples exhibiting atypical responses in the routine Yas’78 MBA protocol, were as a rule also tested the following ways.

(a) The harmonized MBA protocol of the EU Reference Laboratory on Marine Biotoxins (CRL-MBA-SOP, version 5; [42]), which includes a partitioning step between diethylether (DEE) and water. This partitioning step is necessary to confirm the lipophilic nature of a toxin, in case of a positive result. In case of a negative result, however, it could be indicative of interferences due to the presence of hydrophilic toxins (e.g., PSPs), which are quite common and documented for the Yasumoto1978 protocol; and (b) the UPLC-MS/MS in order to confirm the presence of regulated lipophilic biotoxin groups (okadaic acid (OA) group comprising OA, dinophysistoxins (DTXs) and pectenotoxins (PTXs) together, yessotoxin (YTX) group and azaspiracids (AZA) group), as well as non-regulated ones, which can be determined by this method, such as gymnodimines (GYM), spirolides (SPX) and pinnatoxins (PnTXs).

Results of the harmonized MBA protocol tests (injection of DEE fraction) on the four positive samples (1770/2012, 1774/2012, 1786/2012 and 1798/2012) are presented in Table 2. In all four samples, a negative result was obtained, with mice surviving the whole 24-h observation period and either being completely asymptomatic or presenting minor clinical symptoms, such as diarrhea. In one of the samples (1786/2012), the aqueous fraction from the CRL-MBA-SOP protocol was also tested, producing rapid deaths in all three injected mice (<10 min) with severe symptomatology from the nervous system before death. Analysis by UPLC-MS/MS for the presence of lipophilic toxins, on the other hand, showed only trace amounts of OA, GYM, SPX and PnTXs (OA: <20 μg/kg, GYM: <0.2 μg/kg, SPX: <0.2 μg/kg and PnTXs: <0.1 μg/kg), which cannot account for mouse toxicity. The samples were thus designated as negative to the presence of regulated lipophilic toxins and therefore safe for human consumption, in terms of regulatory monitoring. Based on these results, no sanitary measures could be taken by the respective regional competent authority. All the above findings, could not justify the toxicity observed in the Yasumoto 1978 MBA protocol, but also strongly indicated that the toxic substance(s) producing the deaths was most probably of a hydrophilic nature.

The fact that a series of four consecutive samples presented: (a) positive MBA responses in the Yas’78 protocol with similar atypical nervous symptomatology and death times; (b) similar results in the preliminary investigations by the CRL-MBA-SOP protocol and by UPLC-MS/MS or lipophilic toxins; and (c) all coinciding with a bloom of a species bibliographically documented as potentially toxic, at least in a few, even limited, cases, triggered our interest in further investigation of the episode. For this reason, methanolic extracts of all samples and whole tissue of sample 1786/2012 were forwarded to the University of Santiago de Compostela (USC) for cross-checking and further testing. Analysis for the presence of lipophilic toxins by UPLC-MS/MS confirmed the NRLMB findings, similarly detecting the presence of OA, GYM, SPX and PnTXs in trace amounts. On the other hand, analysis by HPLC-FLD for the presence of PSP toxins in sample 1786/2012 revealed the presence of GTX5 at a concentration equivalent to 1.32 μg STX eq/kg, thus not able to account for the presence of toxicity in the MBA. Finally, analysis by LC-MS/MS for the presence of tetrodotoxins by the method of Rodriguez *et al.* [8], resulted in the detection of TTX analogues in that sample and thus a relevant UPLC-MS/MS method for the presence of TTXs was developed in NRLMB to be employed for a full investigation of the episode.

#### 2.1.2. Performance Parameters and Quality Control of the UPLC-MS/MS Method for TTXs

**Figure 2:**
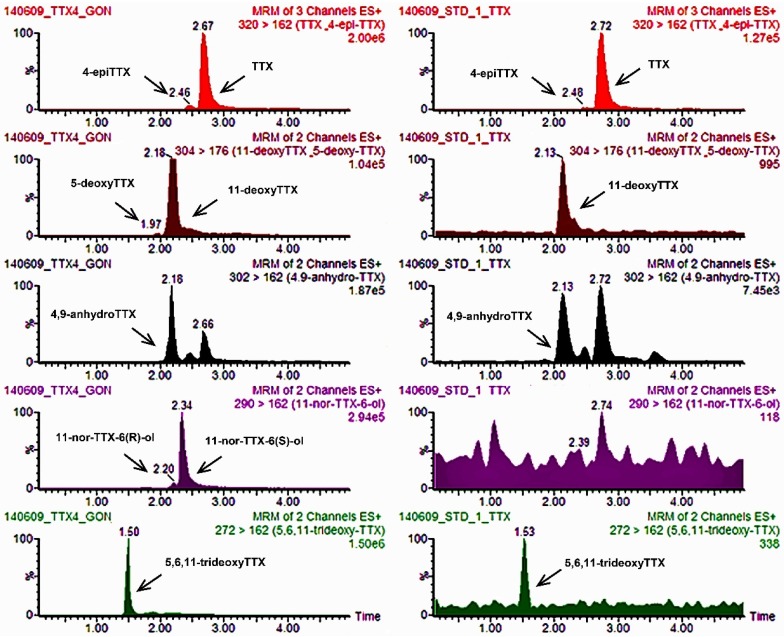
UPLC-MS/MS chromatograms (TIC) of: (**a**) naturally contaminated gonads ample from *L. sceleratus* pufferfish; and (**b**) the certified tetrodotoxins (TTXs) reference standard (TTX = 2000 ng/mL; 4-epiTTX = 49.86 ng/mL; 4,9-anhydroTTX = 467.4 ng/mL). Peak scale has been adjusted in some of the chromatograms to allow presentation of lower concentration peaks.

The certified TTXs (standard containing TTX), 4-epiTTX and 4,9-anhydroTTX, together with naturally contaminated gonad and muscle samples from *Lagocephalus sceleratus* pufferish used in former works [8], provided us with the retention times (RTs) of TTX and its analogues. Values presented for each compound are the mean RT and range from minimum to maximum (in brackets): TTX = 2.73 min (2.56–2.85 min); 4-epiTTX = 2.53 min (2.43–2.65 min); 4,9-anhydroTTX = 2.20 min (2.10–2.34 min); 5,6,11-trideoxyTTX = 1.53 min (1.42–1.61 min); 5-deoxyTTX = 2.02 min (1.92–2.10 min); 11-deoxyTTX = 2.24 min (2.13–2.34 min); 11-norTTX-6(R)-ol = 2.26 min (2.15–2.36 min); and 11-norTTX-6(S)-ol = 2.41 min (2.28–2.49 min) (Figure 2). RTs of TTXs in tested samples when compared to those of the reference standards and naturally contaminated materials were always within the ±2.5% tolerance limit allowed for LC-MS methods in compliance to the requirements of EU Decision 2002/657/EEC [43] for each day of analysis.

**Table 3:** Mean TTX recovery data for mussel digestive gland (DG) and whole flesh (WF) fortified at five concentrations (*n* = 6).

TTX Concentration and Recovery	Tissue	TTX Fortification Level				
		100 μg/kg	200 μg/kg	400 μg/kg	800 μg/kg	1200 μg/kg
Mean TTX detected (μg/kg)	DG	90.1	211.2	431.5	833.9	1171.5
Mean Recovery (%)		90.1	105.6	107.9	104.2	97.6
Mean TTX detected (μg/kg)	WF	94.3	209.3	384.3	754.6	1211.0
Mean Recovery (%)		94.3	104.6	96.1	94.3	100.9

Solutions of TTXs certified reference standard showed excellent linearity within the whole range of standard concentrations for all three analogues contained in the reference standard (TTX, 4,9-anhydroTTX and 4-epiTTX), with R^2^ values higher than 0.995 in all analysis days. The limits of detection (LOD; *S*/*N* > 3) and limits of quantification (LOQ; S/N > 10) of the method obtained from the standard solutions were: TTX = 0.6 and 2.2 ng/mL; 4-epiTTX = 0.7 and 2.3 ng/mL and 4,9-anhydroTTX = 1.9 and 6.5 ng/mL, respectively, corresponding to TTX = 2.2 and 7.2 μg/kg; 4-epiTTX = 2.3 and 7.6 μg/kg and 4,9-anhydroTTX = 6.2 and 21.1 μg/kg in shellfish tissue, respectively.

Trueness of the method, assessed through TTX recovery (%) was within the acceptable levels indicated by the EU Decision 2002/657/EEC [43] in all five levels tested and for both types of tissue (DG or WF) used in the study (Table 3).

**Figure 3:**
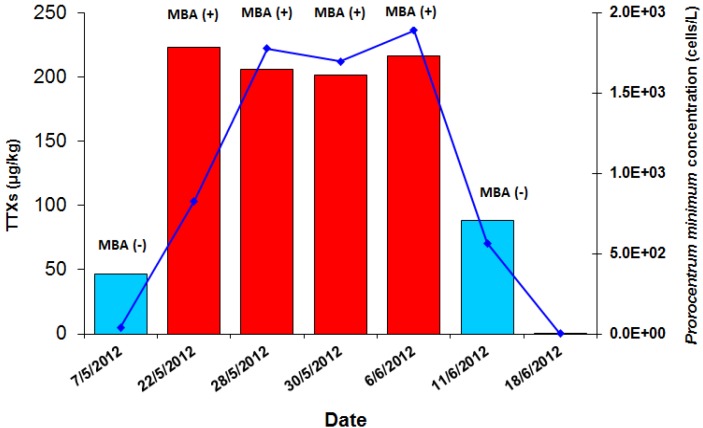
TTXs concentrations (μg/kg) and mouse bioassay (MBA; Yasumoto 1978 protocol) results compared to *Prorocentrum minimum* concentrations (cells/L) of the 2012 shellfish toxicity episode in Vistonikos Bay–Lagos, Rodopi, Greece.

#### 2.1.3. Application of the UPLC-MS/MS Method to the Mussel Samples of the *P. minimum* Episode under Investigation

**Figure 4:**
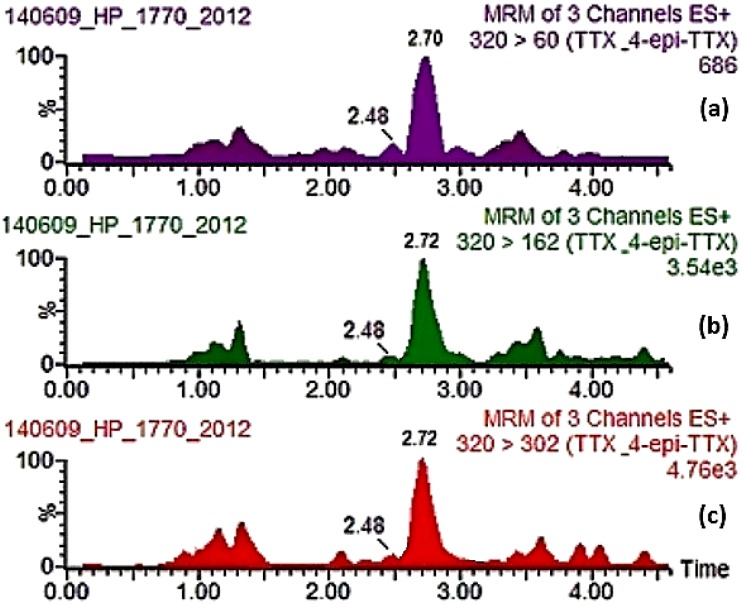
UPLC-MS/MS chromatogram of mussel digestive gland sample 1770/2012 from Vistonikos Bay–Lagos, Rodopi, Greece, containing TTX and 4-epiTTX. (**a**) *m*/*z* 320 > 60; (**b**) *m*/*z* 320 > 162; and (**c**) *m*/*z* 320 > 302.

Results of the UPLC-MS/MS analyses for the presence of TTXs with regard to mussel samples obtained from the episode in question are presented in Table 2. The sum of TTXs concentrations (TTX, 4-epiTTX and 4,9-anhydroTTX) exceeded the level of 200 μg/kg in all four consecutive samples that tested positive in the Yas’78 protocol whereas the sum of TTXs in the two MBA negative ones (one sampled prior and one sampled after the positive ones) were much lower, with the highest concentration being 88.2 μg/kg, possibly coinciding with the decline of the episode. All remaining TTX analogues were either not detected or below the limit of quantification. A quite evident correlation can be observed when these results are graphically compared to the *P. minimum* concentrations recorded during that time period, indicating a possible link between the presence of this potentially toxic species and the presence of TTXs (Figure 3). Figure 4 shows a typical chromatogram of a sample containing TTX.

#### 2.1.4. Experimentally TTX-Contaminated Digestive Gland Tissue Tested by MBA

Results of mouse testing with the Yasumoto1978 protocol on DG tissue experimentally contaminated with TTX at five levels are presented in Table 4. Mouse toxicity was observed down to the fortification level of 100 μg/kg, whereas the 50-μg/kg concentration was non-detectable. Mouse survival times of the experimentally contaminated tissue at the level of 200 μg/kg correlated rather well with those of the MBA positive samples of the 2012 Rodopi episode in which TTXs concentrations were at similar levels.

**Table 4:** Mouse bioassay (MBA) data for blank mussel digestive gland fortified with TTX at five concentrations and tested by the Yasumoto1978 MBA protocol (*n* = 6).

TTX Fortification Level (μg/kg)	Mice Dead	Mouse Survival Time	
		Median	Range
0	0/6	>24 h	>24 h
50	0/6	>24 h	>24 h
100	4/6	310 min	260 min→24 h
200	6/6	85 min	72–98 min
360	6/6	33 min	22–37 min
1000	6/6	9 min	7.5–10 min

### 2.2. Monitoring of Vistonikos Bay–Lagos and Neighboring Coastal Areas in 2014

Due to its previous history with the 2012 aforementioned episode, the coastal area of Vistonikos Bay–Lagos, Rodopi (sampling site Zone 6, Figure 1A) was selected for intensive monitoring of TTXs concentrations for a period of 3 months (8 April to 16 July, 2014), in combination with MBA testing by the Yasumoto1978 protocol and UPLC-MS/MS routine testing for lipophilic toxins, at a weekly basis according to the national monitoring program. The monitoring period was specifically chosen to include the respective time period (end of May–beginning of June) that the previous episode had occurred, in order to also comply with the requirements of Regulation (EC) 15/2011 [41] regarding the “periodic monitoring of production areas and relaying areas for detecting new or unknown marine toxins”. Two samples from the wider coastal area of North Aegean Sea (Strymonikos Gulf, Figure 1C) were also tested for the same purpose. It should be noted that during this period there were no significant occurrences of *P. minimum* indicating a bloom.

MBA results and TTX concentrations of the samples obtained during this 3-month monitoring period are presented in Table 5. During the whole monitoring period, there was only one MBA positive sample (2290/2014) and a negative one (2310/2014) with mice presenting a bad clinical condition. These results were not attributed to the presence of TTXs, as their concentrations were below 50 μg/kg, thus non-detectable by the MBA protocol employed, but could be due to either a synergistic action of the lipophilic toxin groups determined by UPLC-MS/MS analysis in these two samples ((a) sample 2290/2014: OA group = 41.8 μg/kg, YTXs = 0.79 mg/kg, GYMs = 4.8 μg/kg, SPXs = 1.1 μg/kg and PnTX-G = 5.1 μg/kg and (b) 2310/2014: OA group = 51.1 μg/kg, GYMs = 4.7 μg/kg, SPXs = 1.6 μg/kg and PnTX-G = 7.0 μg/kg) or to the presence of other toxins, non-detectable by this method. Nevertheless, it should be noted that low TTXs concentrations (<50 μg/kg) were recorded in all tested samples from this coastal area, indicating a steady presence of this toxin group in shellfish produced in this location.

### 2.3. Investigation of Routine Shellfish Samples from 2006–2012 Obtained in Periods of P. minimum Increased Presence

Reviewing of official control data from the years 2006–2012 identified 17 shellfish samples with atypical positive to asymptomatic negative Yas’78 MBAs for lipophilic toxins, obtained from sampling sites of different Greek production areas (Figure 1) near periods where *P. minimum* blooms or high concentrations (>1000 cells/L) were recorded. UPLC-MS/MS analysis of retained digestive gland or whole flesh sub-samples from these cases already showed the presence of TTXs (TTX, 4-epiTTX and 4,9-anhydroTTX) in Greek shellfish in 2006, in concentrations ranging between 61.0–194.7 μg/kg (Table 6). All remaining TTX analogues were either not detected or below the limit of quantification. A connection of the TTXs concentrations determined with the presence of *P. minimum* could not be shown in these cases, as presence of this potentially toxic species at that time was not steadily reported in terms of the weekly monitoring program. However, the fact that TTXs concentrations in all these cases were higher than 60 μg/kg, in contrast to the results presented in Section 2.2, in combination with the fact that the highest TTXs concentration (194.7 μg/kg) was recorded in an MBA positive sample with death times and symptomatology resembling those of the samples from the 2012 episode in Rodopi (see Section 2.1.3) could point to a possible relationship between *P. minimum* presence in seawater and TTX accumulation in shellfish.

**Table 5:** Sampling data and analysis results of the 2014 three-month monitoring of Vistonikos Bay–Lagos, Rodopi, Greece and neighboring areas.

Sampling Data						MBA (Yasumoto1978)		UPLC-MS/MS				
Year	Sample Code	Date of Collection	Regional Unit	Site	Species	Result (24 h)	Symptomatology	Tissue Tested	TTX (μg/kg)	4-epi-TTX (μg/kg)	4,9-anhydro-TTX (μg/kg)	Sum of TTXs (μg/kg)
2014	2084/2014	08 April 2014	Rodopi	Zone 6	Mussels	Negative (− − −)	None	WF	43.4	ND	ND	43.4
2014	2126/2014	24 April 2014	Rodopi	Zone 6	Mussels	Negative (− − −)	None	WF	38.9	ND	ND	38.9
2014	2141/2014	29 April 2014	Rodopi	Zone 6	Mussels	Negative (− − −)	None	WF	34.9	ND	ND	34.9
2014	2167/2014	07 May 2014	Rodopi	Zone 6	Mussels	Negative (− − −)	None	WF	37.7	ND	ND	37.7
2014	2172/2014	08 May 2014	Rodopi	Zone 6	Mussels	Negative (− − −)	None	WF	34.8	ND	ND	34.8
2014	2203/2014	20 May 2014	Rodopi	Zone 6	Mussels	Negative (− − −)	None	WF	30.1	ND	ND	30.1
2014	2239/2014	28 May 2014	Rodopi	Zone 6	Mussels	Negative (− − −)	2/3 mice with mild diarrhea	WF	25.8	ND	ND	25.8
2014	2232/2014	28 May 2014	Serres	S1: Nea Kerdyllia	Mussels	Negative (− − −)	None	WF	37.9	9.1	ND	47.0
2014	2234/2014	28 May 2014	Chalkidiki	X2: South Strymonikos Gulf—Olympiada	Mussels	Negative (− − −)	None	WF	30.6	<LOQ (3.2) *	ND	33.8
2014	2270/2014	11 June 2014	Rodopi	Zone 6	Mussels	Negative (− − −)	3/3 mice with diarrhea	WF	28.4	ND	ND	28.4
2014	2290/2014	19 June 2014	Rodopi	Zone 6	Mussels	Positive (− + +)	1/3 mice dead in 3:30–19:30 h, 1/3 dead in 21:30–22:00 h	WF	36.8	ND	ND	36.8
2014	2310/2014	25 June 2014	Rodopi	Zone 6	Mussels	Negative (− − −)	3/3 mice with bad clinical condition	WF	25.1	ND	ND	25.1
2014	2319/2014	02 July 2014	Rodopi	Zone 6	Mussels	Negative (− − −)	3/3mice with bad clinical condition and diarrhea	WF	33.3	ND	ND	33.3
2014	2336/2014	08 July 2014	Rodopi	Zone 6	Mussels	Negative (− − −)	3/3 mice with diarrhea	WF	25.0	ND	ND	25.0
2014	2362/2014	16 July 2014	Rodopi	Zone 6	Mussels	Negative (− − −)	None	WF	37.6	ND	ND	37.6

MBA = Mouse bioassay, TTX = tetrodotoxin, WF = whole flesh, ND = not detected. Values with an asterisk indicate concentrations between the calculated method LOD and LOQ and are shown as contributions to the sum of TTXs.

**Table 6:** Sampling data and analysis results of routine shellfish samples from 2006–2012 obtained in periods of *P. minimum* presence.

Sampling Data						*Prorocentrum minimum* (cells/L)	MBA (Yasumoto1978)		UPLC-MS/MS				
Year	Sample Code	Date of Collection	Regional Unit	Site	Species		Result (24 h)	Symptomatology	Tissue Tested	TTX (μg/kg)	4-epi-TTX (μg/kg)	4,9-anhydro-TTX (μg/kg)	Sum of TTXs (μg/kg)
2006	1357/2006	20 March 2006	Dytiki Attiki/ Megara	Drepano	Mussels	No data provided	Positive (+ + +)	3/3 mice dead in <15 h	DG	70.7	<LOQ (6.7) *	ND	77.4
2006	1366/2006	11 April 2006	Dytiki Attiki/ Megara	Neraki	Mussels	3.50 × 10^4^	Positive (+ + −)	2/3 mice dead in <15 h	DG	68.4	<LOQ (3.4) *	ND	71.8
2006	1367/2006	11 April 2006	Dytiki Attiki/ Megara	Drepano	Mussels	7.50 × 10^4^	Negative (+ − −)	1/3 mice dead in <15 h, 1/3 mild diarrhea	DG	61.2	<LOQ (7.5) *	ND	68.8
2008	1547/2008	9 September 2008	Dytiki Attiki/ Megara	Drepano	Mussels	7.50 × 10^4^	Positive (+ + −)	1/3 mice dead in 2:00–12:30 h, 1/3 in 23:00–23:45 h, 1/3 with bad clinical condition and diarrhea (and death in 26:00–36:30 h)	DG	55.4	<LOQ (4.7) *	<LOQ (12.4) *	72.5
2008	1583/2008	6 October 2008	Dytiki Attiki/ Megara	Neraki	Mussels	No data provided. Bloom in previous month	Negative (− − −)	3/3 mice with bad clinical condition and diarrhea (and death 2/3 in 26:00–35:30 h and 1/3 in 42:30–44:00 h)	DG	52.9	<LOQ (4.3) *	<LOQ (10.8) *	68.0
2008	1590/2008	13 October 2008	Dytiki Attiki/ Megara	Neraki	Mussels	No data provided. Bloom in previous month	Positive (+ + +)	1/3 mice dead in 2:00–2:30 h, 1/3 in 2:20–2:40 h, 1/3 in 2:40–3:30 h	DG	65.6	<LOQ (4.7) *	<LOQ (15.1) *	85.4
2008	1594/2008	14 October 2008	Attiki/Nisson	Salamina—Zone Ι—Vasilika–Faneromeni	Venus clams	Bloom in the wider area during the previous month (Neraki, Drepano)	Positive (+ + +)	3/3 mice dead in 2:00–2:30 h	DG	176.5	<LOQ (3.9) *	<LOQ (14.3) *	194.7
2008	1607/2008	23 October 2008	Dytiki Attiki/ Megara	Neraki	Mussels	3.68 × 10^5^	Negative (− − −)	2/3 bad clinical condition and diarrhea, 1/3 bad clinical condition and mild diarrhea	DG	62.6	<LOQ (4.9) *	<LOQ (18.6) *	86.2
2008	1625/2008	4 November 2008	Attiki/Nisson	Salamina—Zone ΙV—Agios Georgios	Mussels	Simultaneous bloom in the wider area (Neraki: 1.11 × 10^4^)	Negative (− − −)	1/3 mice with bad clinical condition and diarrhea, 2/3 bad clinical condition and mild diarrhea (and death 1/3 in 27:35–37:10 h and 1/3 in 45:00–46:20 h)	DG	57.2	<LOQ (3.8) *	ND	61.0
2009	1544/2009	16 June 2009	Rodopi	Zone 6	Mussels	Bloom in the previous month (6 May 2009: 1.13 × 10^5^ & 12 May 2009: 8.24 × 10^3^)	Positive (+ + +)	2/3 mice dead in 13:00–21:00 h, 1/3 in 21:00–23:00 h	DG	46.7	<LOQ (5.4) *	<LOQ (19.2) *	71.3
2009	1552/2009	22 June 2009	Rodopi	Zone 6	Mussels	No data provided	Negative (− − −)	3/3 mice with bad clinical condition and diarrhea (and death 3/3 in 29:20–46:20 h)	DG	48.1	<LOQ (5.0) *	<LOQ (15.9) *	69.0
2009	1556/2009	24 June 2009	Rodopi	Zone 6	Mussels	No data provided	Positive (+ + −)	2/3 mice dead in 1:30–2:00 h, 1/3 with bad clinical condition and diarrhea (and death in 30:00–48:00 h)	DG	52.6	<LOQ (3.5) *	<LOQ (8.6) *	64.8
2012	2057/2012	6 November 2012	Rodopi	Zone 6	Mussels	No data provided. Bloom reported in the following 2 weeks, max. 5.36 × 10^5^	Negative (− − −)	2/3 mice with bad clinical condition and mild diarrhea, 1/3 mice with bad clinical condition	DG	68.5	<LOQ (3.4) *	<LOQ (12.0) *	83.9
2012	1216/2012	22 November 2012	Kavala	Keramoti	Mussels	No data provided. Bloom reported in the following weeks	Positive (+ − +)	2/3 mice dead in 4:00–20:00 h, 1/3 mice with bad clinical condition	DG	71.0	<LOQ (3.4) *	<LOQ (20.7) *	95.0
2012	1224/2012	10 December 2012	Kavala	Keramoti	Mussels	3.13 × 10^4^	Negative (− − +)	1/3 mice dead in 2:00–18:00 h, 2/3 mice with bad clinical condition and mild diarrhea	DG	71.2	<LOQ (5.2) *	<LOQ (11.7) *	88.2
2012	1227/2012	17 December 2012	Kavala	Keramoti	Mussels	1.56 × 10^4^	Negative (− − −)	2/3 mice with bad clinical condition and mild diarrhea, 1/3 mice with bad clinical condition	DG	91.8	<LOQ (4.3) *	<LOQ (13.2) *	109.2
2012	1241/2012	3 December 2012	Thessaloniki	Kymina	Mussels	174 (in wider area 1.75 × 10^5^)	Negative (− − −)	None	WF	79.5	<LOQ (5.6) *	<LOQ (18.0) *	103.1

MBA = Mouse bioassay, TTX = tetrodotoxin, DG = digestive glands, WF = whole flesh, ND = not detected. Values with an asterisk (*) indicate concentrations between the calculated method LOD and LOQ and are shown as contributions to the sum of TTXs.

**Table 7:** Sampling data and analysis results of random routine shellfish samples obtained in June–July 2014.

Sampling Data						MBA (Yasumoto1978)		LC-MS/MS			
Year	Sample Code	Date of Collection	Regional Unit	Site	Species	Result (24 h)	Symptomatology	Tissue Tested	TTX (μg/kg)	4-epi-TTX (μg/kg)	4,9-anhydro-TTX (μg/kg)
2014	2303/2014	23 June 2014	Chalkidiki	X2: South Strymonikos Gulf—Olympiada	Mussels	Negative (− − −)	3/3 mice with diarrhea	HP/WF	<LOQ	ND	ND
2014	272/2014	30 June 2014	Thessaloniki	Kymina	Mussels	Negative (− − −)	None	HP/WF	ND	ND	ND
2014	2313/2014	30 June 2014	Dytiki Attiki/ Megara	Drepano	Mussels	Negative (− − −)	3/3 mice with bad clinical condition and diarrhea	HP	ND	ND	ND
2014	2315/2014	30 June 2014	Dytiki Attiki/ Megara	Neraki	Mussels	Negative (− − −)	None	HP/WF	ND	ND	ND
2014	1303/2014	01 July 2014	Kavala	Keramoti	Mussels	Negative (− − −)	None	HP/WF	<LOQ	ND	ND
2014	2323/2014	01 July 2014	Serres	S1: Nea Kerdyllia	Mussels	Negative (− − −)	None	HP/WF	<LOQ	ND	ND
2014	283/2014	07 July 2014	Thessaloniki	Kymina	Mussels	Negative (− − −)	1/3 mice with mild diarrhea	HP/WF	ND	ND	ND
2014	2332/2014	07 July 2014	Dytiki Attiki/ Megara	Drepano	Mussels	Negative (− − −)	3/3 mice with bad clinical condition	HP	ND	ND	ND
2014	2334/2014	07 July 2014	Dytiki Attiki/ Megara	Neraki	Mussels	Negative (− − −)	None	HP/WF	ND	ND	ND
2014	2337/2014	07 July 2014	Chalkidiki	X2: South Strymonikos Gulf—Olympiada	Mussels	Negative (− − −)	1/3 mice with bad clinical condition	HP	<LOQ	ND	ND
2014	1307/2014	08 July 2014	Kavala	Keramoti	Mussels	Negative (− − −)	None	HP	ND	ND	ND
2014	2342/2014	08 July 2014	Serres	S1: Nea Kerdyllia	Mussels	Negative (− − −)	1/3 mice with diarrhea	HP	ND	ND	ND
2014	297/2014	14 July 2014	Thessaloniki	Kymina	Mussels	Negative (− − −)	1/3 mice with diarrhea, 2/3 mild diarrhea	HP	ND	ND	ND
2014	1311/2014	15 July 2014	Kavala	Keramoti	Mussels	Negative (− − −)	1/3 mice with bad clinical condition	HP	ND	ND	ND
2014	2360/2014	15 July 2014	Dytiki Attiki/ Megara	Drepano	Venus clams	Negative (− + −)	1/3 mice dead in 1:05 h, 1/3 diarrhea	HP/WF	ND	ND	ND
2014	2368/2014	15 July 2014	Serres	S1: Nea Kerdyllia	Mussels	Negative (− − −)	None	HP/WF	ND	ND	ND

### 2.4. Investigation of Random Routine Shellfish Samples Obtained in June–July 2014

Digestive gland and whole flesh samples from a number of randomly selected samples obtained in June and July 2014 from the same areas (except Rodopi) where previous presence of TTXs was recorded (see Section 2.2 and Section 2.3) were also analyzed by UPLC-MS/MS for the presence of TTXs (Table 7). TTXs were not detectable or below the limit of quantification in these samples, some of which served as negative tissue material for the fortification experiments of the study.

## 3. Discussion

### 3.1. UPLC-MS/MS Method Performance

TTXs reference standard solutions exhibited excellent linearity in the developed UPLC-MS/MS method for the determination of TTXs over the whole calibration ranges of all three analogues, with R^2^ values higher than 0.995 for all analysis days. Mean TTX recovery at the five fortification levels (100, 200, 400, 800 and 1200 μg/kg) selected in the present study and for both tested matrices (mussel DG and WF) was good, ranging between 90.1% and 107.9%. These values are in compliance with the 90%–110% range indicated as acceptable recovery levels according to the requirements of EU Decision 2002/657/EEC [43], when recovery is assessed by fortification.

### 3.2. Presence of TTXs in Greek Shellfish

In the present study, concentrations of the sum of TTXs in a total of 38 shellfish samples selected for UPLC-MS/MS analysis ranged from 25.0 μg/kg up to a maximum of 222.9 μg/kg. TTX was the most abundant of all TTXs and was present in all 38 samples, at concentrations ranging from 25.0 to 202.9 μg/kg. The analogues 4-epiTTX and 4,9-anhydro TTX were quantified in lower concentrations, in 24 and 19 out of 38 samples, respectively. On the other hand, 5,6,11-trideoxyTTX and the remaining analogues were not found at quantifiable concentrations, a fact attributable to either their absence in our samples, as they probably result by metabolism in shellfish [13], or to possible differences in method sensitivity for these compounds. It should also be noted that a number of mussel samples negative to the presence of TTXs were found among the routine samples of regulatory monitoring (data not shown) and these were used for the fortification experiments conducted in terms of method recovery assessment.

The TTXs levels of our study are substantially higher than those recently reported by Turner *et al.* [13] in bivalve mollusks (blue mussels and pacific oysters) harvested in 2013–2014 from the south coast of England, along the Channel. This is expectable, taking into account that TTX is traditionally occurring in warmer tropical or subtropical waters and that Greece is located more southern compared to England, with a substantially warmer climate. Furthermore, the present work is mostly based on analyses of targeted and specifically selected samples, either connected to the presence of *P. minimum* blooms or derived from coastal areas with a previous history of TTXs present in increased levels.

Various TTXs levels have been reported in gastropods and other bivalve species in Europe and internationally. Rodriguez *et al.* [12] found very high TTXs concentrations (TTX: 315 mg/kg and 5,6,11-trideoxyTTX: 1004 mg/kg) in the digestive glands of the trumpet shell *Charonia lampas lampas* sample harvested from the south coast of Portugal and purchased from Malaga market. Such increased TTXs concentrations in this specific sample, however, are not common but were expectable due to the fact that this sample was implicated in a food poisoning incident. Indeed, Nzoughet *et al.* [44] reported much lower TTX levels of 22.4 and 66.6 μg/kg in viscera and muscle, respectively, of naturally contaminated trumpet shells from the same species, harvested from Angeiras Coast, Portugal. Similarly, the presence of TTXs at low levels was also reported in two more gastropod species harvested from Portuguese coasts: *Gibbula umbilicalis* (monodeoxyTTX: 63.81 μg/kg) and *Monodonta lineata* (TTX: 90 μg/kg and 4-epiTTX: 21 μg/kg) [45]. The only other reported occurrences of TTX in bivalve mollusks are those by McNabb *et al.* [10],who found TTX in the New Zealand clam *Paphies australis* at levels up to 800 μg/kg and by Kodama *et al.* [11] who reported the presence of TTX in the Japanese scallop *Patinopecten yessoensis*.

Our study constitutes, to our best knowledge, the earliest report (March 2006) for TTXs detection in European bivalve mollusks, mussels and venus clams, as well as the first report for shellfish harvested in the Mediterranean Sea. Although the TTXs levels found in our samples are relatively low when compared to other species implicated in human intoxication incidents, the possibility of increase in their concentrations in the near future cannot be excluded. TTXs presence in bivalves is not routinely monitored or regulated in Europe or anywhere else in the world, given the absence so far of published data demonstrating a risk of TTX intoxication from bivalves. In the present study, TTX presence was revealed due to positive results and atypical responses in the MBA for lipophilic toxins. Until mid-2011, this situation would result in a regulatory closure of the affected area, thus the consumer would be protected from exposure to this toxin. This is not anymore the case, as due to the replacement of MBA by LC-MS/MS as a reference method for the presence of lipophilic toxins, such “false positive” results are not detectable, thus exposing the consumers to TTX and analogues together with all possible toxins not detectable by the currently existing routine testing methods.

Taking into account the absence of any formal regulatory guidance for the presence of TTX in shellfish, the maximum concentration of 222.9 μg/kg TTX in the present study, equates to *ca.* 28% of the maximum permitted level for saxitoxin (STX) equivalents (800 μg STX equivalents/kg shellfish tissue), noting the similarity in biological activity between the two toxin groups. The TTXs level of 222.9 μg/kg would also equate to a low level dose of toxin in comparison to the proposed minimum lethal dose (MLD) for TTX of between 0.5 to 2 mg [19], but would be quite near to the minimum acute dose (0.2 mg) for induction of symptomatology to humans (wt. 50 kg) [3]. In the same context, consumption of 500 g of shellfish contaminated with 222 μg/kg of TTXs would equate to the intake of *ca.* 110 μg TTX, *i.e.*, almost22% of the proposed MLD if taken as 0.5 mg TTX for a 60 kg human [46]. Such rough calculations, of course, do not incorporate any additional safety factors as applied by the European Food Standards Agency (EFSA) in their risk assessment methods, taking into account measurement or toxicity-related uncertainties [47], and/or the possibility of high toxin content variability in bulk samples of shellfish across harvesting areas.

Consequently, while the human health risk arising from the specific samples analyzed in our study is rather low, there is the potential for health impacts, especially if TTX levels were significantly higher at other times or in other areas associated with shellfish harvesting. It is important to note that while bacterial pathogens may be eliminated in shellfish products following effective cooking, TTXs are heat stable and will thus not be destroyed in the food preparation process [13].

### 3.3. Comparison of Tetrodotoxin to Venerupin Shellfish Poisoning Toxin

Unfortunately no unpreserved seawater samples containing alive *P. minimum* cells were available in order to obtain cultures of the specific strain causing the 2012 episode in Rodopi, which would allow an in-depth study of its toxin production potential. Certain results from the present study, however, combined with published findings from previous works, point to the suspicion that Venerupin Shellfish Poisoning (VSP) toxin associated with *P. minimum* blooms could be related to tetrodotoxin. Relevant facts supporting this hypothesis would be: (a) the increase and decline of TTXs concentrations in mussels of the 2012 episode in Rodopi almost in parallel with increase and decline of *P. minimum* concentrations (Table 2 and Figure 3); (b) the presence of TTXs in a number of samples selected according to instances of *P. minimum* blooms at levels higher than 60 μg/kg and up to a maximum of 194.7 μg/kg (Table 6); and (c) the presence of TTXs at much lower levels (<50 μg/kg) in a coastal area with a previous history of TTXs presence in increased levels, when no blooms of *P. minimum* were recorded (Table 5).

Where data available in the bibliography are concerned, important facts include: (a) previous incrimination of *P. minimum* for several episodes of human poisoning subsequent to the consumption of shellfish, with a symptomatology characteristic of paralytic shellfish poisoning (PSP) [26,27,28,29], which is rather difficult to distinguish with that of TTX poisoning [19]; (b) mussel toxicity associated to the presence of *P. minimum* by a water soluble neurotoxic component, different to PSP but also inhibiting calcium channels, that rapidly killed mice [37]; (c) the subsequent isolation of the same toxic fraction from shellfish during a large *P. minimum* [38]; and (d) the strain specific and stimulated by associated bacteria character of the toxin production by *P. minimum* [37], also taking into account the generally accepted bacterial origin of tetrodotoxin. All these findings are in agreement with our observations with regard to the hydrophilic and neurotoxic nature of the compound causing shellfish toxicity in the 2012 episode in Vistonikos Bay, where TTX was detected. However, it would be rather premature to conclude that TTX and VSP are indeed identical, as a number of experiments would be necessary to verify this hypothesis. Upon occurrence of a future *P. minimum* toxic episode, therefore, further research will have to be undertaken, including obtaining seawater field samples, establishing cultures of the specific strain and TTX analysis of the experimental materials in order to elucidate the actual mode of *P. minimum* toxin production and to establish the exact, if any, role of this species regarding the presence of TTXs in shellfish.

## 4. Experimental Section

### 4.1. Standards and Reagents

A certified reference standard for tetrodotoxins (CRM-03-TTXs; purity >96%), purified from puffer fish, was obtained from Laboratorio CIFGA S.A. (Lugo, Spain). The standard has a certified reference concentration of 25.7 ± 2.1 μg/mL for tetrodotoxin (TTX) and 3.0 ± 0.4 μg/mL for 4,9-anhydro tetrodotoxin (4,9-anhTTX) and a non-certified reference concentration of 0.32 ± 0.08 μg/mL for 4-epi tetrodotoxin (4-epiTTX). Certified reference standards for lipophilic toxins analysis (okadaic acid (NRC-CRM-OA-c), yessotoxin (NRC-CRM-YTX), pectenotoxin-2 (NRC-CRM-PTX2), azaspiracid-1 (NRC-CRM-AZA1), gymnodimine-A (NRC-CRM-GYM) and 13-desmethyl-spirolide C (NRC-CRM-SPX1)), were purchased from the National Research Council of Canada (Halifax, Canada). The pre-certified reference standard for pinnatoxin-G (RM-PnTX-G) was a generous gift from Pearce McCarron (NRC, Halifax, Canada). Acetic acid was of analytical grade, methanol of HPLC grade, acetonitrile and water of LC-MS grade, whereas formic acid and ammonium formate were of mass spectrometry grade. Chemicals were obtained from Fisher Scientific UK (Loughborough, UK), Panreac (Barcelona, Spain), Fluka (Sigma Aldrich group, Schnelldorf, Germany) and Alfa Aesar (Karlsrube, Germany).

### 4.2. Sampling and Test Materials

Samples and respective data on *P. minimum* counts and presence of lipophilic toxins were derived from the Greek “National Programme for Monitoring of Bivalve Molluscs” Production Areas for the presence of Marine Biotoxins” during the years 2006–2014; coordination was provided by the Ministry of Productive Reconstruction, Environment and Energy (former Ministry of Rural Development and Food [48,49]). All samplings were conducted by the relevant Prefectural Veterinary and/or Fisheries authorities. Cell counts of toxic and/or potentially toxic phytoplankton in seawater, and specifically of the species *P. minimum*, were conducted by the Laboratory Unit of Toxic Marine Microalgae (LUTMM), Department of Biology, Aristotle University of Thessaloniki (scientific coordinator: G. Nikolaidis (until February 2010) and M. Arsenakis (March 2010–to date), and data were forwarded to the NRLMB initially by the prefectural authorities and later on by the conducting laboratory, as indicated by the monitoring program requirements. Mussel (*Mytilus galloprovincialis*) samples and additional seawater samples, in the case of the main investigated episode of Rodopi were collected from the sampling site “Zone 6” in Vistonikos Gulf, North Aegean Sea (Figure 1). The shellfish samples tested from previous years (mussels: *Mytilus galloprovincialis* and clams: *Venus verrucosa*) were obtained from various sampling sites along the Greek coasts. Random samples from these latter sites, obtained in June and July 2014, were also tested in order to check TTXs background levels in these areas and to provide negative tissue material for fortification experiments (see Figure 1 and Table 1, Table 5, Table 6 and Table 7).

### 4.3. Seawater Analyses

Seawater samples were analyzed for the presence of toxic and/or potentially toxic phytoplankton by Utermöhl’s sedimentation method [50], using inverted microscopy for identification and enumeration of the phytoplankton cells. Briefly, after thorough homogenization, sub-samples were allowed to settle on 25 mL sedimentation chambers. Cells were counted in an inverted microscope, at 100× and 400× magnifications. It should be noted that until November 2012, individual concentrations of *P. minimum* cells were not regularly reported by the testing laboratory (LUTMM), as they were not perceived as potentially toxic in the context of the bivalve mollusks’ monitoring program, but only mentioned as responsible for ichthyotoxicity. For this reason, additional seawater samples obtained for investigation of the 2012 Rodopi episode had to be enumerated *ad hoc* during the episode development, in order to obtain a full dataset with regard to *P. minimum* presence for each sample obtained at that period.

### 4.4. Shellfish Preparation and Sub-Sampling

Shellfish samples were washed and shucked upon arrival to the laboratory; digestive glands (DG) and whole flesh (WF) were separated and homogenized. All samples were tested for the presence of lipophilic toxins by mouse bioassay (MBA), according to the protocol of Yasumoto *et al.* (1978) [39] (see Section 4.5), whereas a number of samples were also further investigated using the EU-harmonized MBA protocol of the European Reference Laboratory on Marine Biotoxins (version 5; [42]). According to the NRLMB laboratory practice, DG and/or WF sub-samples were retained for each tested sample, for at least one year after the analysis. However, in the case of samples with positive results or negative results with severe symptomatology, with an emphasis being paid on atypical responses, sub-samples are retained as a rule for indefinite time under deep freezing conditions (−70°C),for the purpose of further investigation in due time.

### 4.5. Mouse Bioassay Tests

Mussel DG samples (routine and fortified with TTX at various levels) were tested for the presence of lipophilic toxins by the mouse bioassay (MBA) protocol of Yasumoto *et al.* (1978). Briefly, a 20 g portion of DG was extracted thrice with 50 mL acetone each time and filtered through a cellulose filter. The combined toxin extract was evaporated to dryness and resuspended in 1% Tween-60 to a final volume of 4 mL (5 g DG/mL). Each one of three mice (Albino Swiss, 18–20 g body weight) was injected intraperitoneally with 1 mL of this solution.

Where further investigation was required, mussel whole flesh samples were also tested for the presence of lipophilic toxins using the harmonized EU-MBA protocol (version 5; [42]). An aliquot of 100 g of whole flesh tissue homogenate was weighed into 500 mL plastic centrifuge containers (Sigma Laborzentrifugen GmbH, Osterode am Harz, Germany), to which acetone (300 mL) was added. The mixture was homogenized for 2 min using an Ultra turrax (15 mm shaft, 10,000 rpm; IKA, Staufen, Germany) and centrifuged at 1200× *g* for 10 min at 4 °C (Centrifuge Sigma 4K15C; Sigma Laborzentrifugen GmbH, Osterode am Harz, Germany). The supernatant was transferred through a filter paper into a labeled 1 L round-bottom flask, while the tissue residue was re-extracted in the same way with 200 mL of acetone. The combined 500 mL filtrate was rotary evaporated under vacuum at 42 °C ± 2 °C (Rotavapor R-200, Buchi, Flawil, Switzerland), until complete removal of acetone. The aqueous residue of the round-bottom flask was transferred to a 100 mL glass cylinder and volume was adjusted to 100 mL by addition of de-ionized water, while 100 mL of diethyl ether were used to rinse off the residue of the round bottom flask. Both aqueous and diethyl ether extracts were transferred to a 500 mL separatory funnel. The mixture was agitated and following separation of the aqueous and diethyl ether phases, the aqueous layer was transferred back into the round-bottom flask. The water was re-extracted twice with 100 mL of diethyl ether and separated in the separatory funnel yielding *ca.* 300 mL of diethyl ether extract, which was backwashed twice with 20 mL de-ionized water. The ether phase was collected in another round-bottom flask and rotary evaporated to dryness under vacuum at 42 °C ± 2 °C. The dry residue was resuspended in 1% Tween-60 to a final volume of 4 mL (25 g whole flesh/mL). When necessary, the resuspended extract was further homogenized using an Ultra turrax (8 mm shaft) prior to injection. Each one of three mice (Albino Swiss, 18–20 g body weight) was injected intraperitoneally with 1 mL of this solution.

In both protocols, the criterion of toxicity established by the EU Regulation 2074/2005 (2005) was employed, which is the death of two out of three mice within 24 h of injection with an extract equivalent to 5 g of DG or 25 g of whole flesh tissue. This constitutes a positive result for the presence of the lipophilic toxins mentioned in the above Regulation. The mice were allowed laboratory feed and water *ad libitum* throughout the observation period. All animal manipulations were performed in accordance with the EU Directive 86/609/EEC (1986) and the EU Recommendation 2007/526/EC (2007), under official license from the Prefectural Veterinary Service of Thessaloniki, Greece (licensed facilities: for conduction of MBAs no. EL54BIO07 and for breeding mice no. EL54BIO08).

### 4.6. UPLC-MS/MS Analysis (Ultra-Performance Liquid Chromatography Coupled to Tandem Mass Spectrometry)

#### 4.6.1. Sample Treatment

Aliquots of 2 g of sample tissue (DG or WF) were extracted in 6 mL of 1% acetic acid in methanol by vortex mixing for 5 min (Multi Reax, Heidolph Instruments, Schwabach, Germany), then further homogenized for 1 min using an Ultra turrax (18 mm shaft, 24,000 rpm; IKA, Staufen, Germany) and ultrasonic waterbath (10 min, 40 kHz) (T910DH, Elma Transsonic Digitals, Singen, Germany). The tubes containing the homogenized extracts were tightly closed with parafilm^®^ (3M) to prevent solvent evaporation and were placed in a waterbath for 10 min at 100°C. After cooling to room temperature, volume was adjusted to 7.5 mL, where necessary, with the methanolic acetic acid solution and extracts were centrifuged at 3000× *g* for 10 min at 4°C (4K15C, Sigma Laborzentrifugen GmbH, Osterode am Harz, Germany). The supernatants were filtered through 0.20 μm methanol compatible nylon syringe filters (Titan 3, Thermo Scientific, Nashville TN, USA) and were placed in vials for UPLC-MS/MS analysis.

#### 4.6.2. UPLC-MS/MS Method

An Acquity UPLC system coupled to a TQD mass spectrometer (Waters, Manchester, UK) equipped with a Z-Spray ESI source was used for sample analysis. The UPLC system was equipped with an Acquity UPLC BEH Amide column (2.1 × 150 mm, 1.7 μm) and an Acquity UPLC BEH VanGuard Pre-Column (2.1 × 5 mm, 1.7 μm, 130 Å), with column oven maintained at 25 °C. The mobile phase consisted of 100% water in channel A and 95% acetonitrile (ACN) in channel B, both containing 2 mM ammonium formate and 50 mM formic acid. An isocratic elution was used with a flow rate of 0.4 mL/min at a ratio of 35% A and 65% B for a total of 5 min and the injection volume was 10 μL (partial loop with needle overfill mode). The mass spectrometer operated in electrospray positive mode with the capillary voltage set at 2.8 kV. The TQD mass spectrometer operated with the following optimized source-dependent parameters (ESI source): capillary voltage 2.8 kV, cone voltage 40 V, desolvation temperature 350 °C, desolvation gas flow 800 L/h N_2_, cone gas flow 50 L/h N_2_, source temperature 120 °C, collision gas flow 0.10 mL/min Argon. The cone voltage and collision energy were optimized for TTX by standard infusion. MassLynx 4.1 with QuanLynx software (Waters, Manchester, UK) was used for data processing.

The mass spectrometer operated in MRM, detecting in positive mode, analyzing at least two product ions per compound: one for quantification (the most abundant) and another for confirmation. The transitions employed were: TTX and 4-epiTTX (*m*/*z* 320 > 302/162/60), 4,9-anhTTX (*m*/*z* 302 > 256/162), 5,6,11-trideoxyTTX (272 > 254/162), 11-deoxyTTX and 5-deoxyTTX (304 > 286/176) and 11-norTTX-6(S)-ol and 11-norTTX-6(R)-ol (290>272/162) and were selected according to Yotsu-Yamashita *et al.* [51], Rodriguez *et al.* [8] and McNabb *et al.* [10] following optimization, where possible, by infusion of reference standards.

For the calibration curve, serial dilutions from the certified TTXs stock standard were performed using 1% acetic acid in methanol to provide eight calibration standards. The concentration ranges were: TTX: 15.63–2000 ng/mL, 4-epiTTX: 0.39–49.86 ng/mL and 4-9-anhTTX: 3.65–467.4 ng/mL. These compounds were quantified using their peak areas to calculate amounts and using the relevant curve obtained from the standard, while the remaining analogues were quantified using the TTX curve and assuming an equal molar response factor. TTXs concentrations in samples were expressed in μg/kg. The calibration range was chosen in order to include concentrations of TTX currently found in various aquatic species [7,8,12,13,44,45,52,53,54] and also to encompass the EU regulatory limit (800 μg/kg) for PSP toxins set by Regulation (EC) no 853/2004 [55]. To overcome the challenge of the lack of standards for some of the TTX analogues, gonad and muscle samples of a naturally-contaminated *Lagocephalus sceleratus* pufferish used in former works [8] were injected in the UPLC-MS/MS, this way determining the respective retention times (RT) (Figure 2).

#### 4.6.3. UPLC-MS/MS Method Quality Control

Negative mussel DG and WF tissues (*i.e.*, free from TTX) were fortified at five concentrations, *i.e.*,100, 200, 400, 800 and 1200 μg/kg, equivalent to, respectively, 0.125, 0.25, 0.5, 1 and 1.5 times the regulatory limit for PSP toxins for the purpose of evaluating the method performance. Fortified materials were extracted following the extraction procedure described in Section 4.6.1 with six independent replicates for each concentration. The mean method recovery for each concentration and tissue was calculated. Linearity of calibration curves, as well as limit of detection (LOD) and limit of quantification (LOQ) based on signal to noise ratios for all three analogues included in the certified TTXs standard were also assessed.

## 5. Conclusions

The present work, to our knowledge, constitutes the earliest report (March 2006) for the presence of TTXs in European bivalve mollusks, mussels and venus clams, as well as the first report for shellfish harvested in the Mediterranean Sea. Taking into account the evidence presented for TTXs occurrence in European bivalve mollusks, and the traditional occurrence of these toxins in warm tropical waters, an important question is whether this is linked to climate change. TTXs have until recently been assumed not to occur in bivalve mollusks, particularly in temperate waters. Since these bivalve species are largely consumed, the probability of human health hazards seems to be increasing. Therefore, despite the low concentrations detected, ranging from 25.0 to 222.9 μg/kg, it is evident that the presence of TTX and analogues should be monitored in all species that can potentially accumulate the toxins and can be used as human food, especially in combination with monitoring of *P. minimum* blooms.

In the present work, preliminary indications were found for the existence of a possible link between *P. minimum* blooms and presence of TTXs in shellfish. Although reported association between *P. minimum* blooms and human toxicity are rare, further research is required, when another episode of *P. minimum* combined with shellfish toxicity re-occurs in the future, in order to elucidate the mode of *P. minimum* toxin production and to establish the exact, if any, role of this species regarding the presence of TTXs in shellfish.
